# An international consensus on core reproducibility items in research

**DOI:** 10.1371/journal.pbio.3003726

**Published:** 2026-04-16

**Authors:** Rita Banzi, Monika Varga, Yuri Andrei Gelsleichter, Constant Vinatier, David Moher, Florian Naudet, on behalf of the OSIRIS-Delphi Study Group

**Affiliations:** 1 Laboratory for Health Regulatory Policies, Mario Negri Institute for Pharmacological Research IRCCS, Milan, Italy; 2 Institute of Animal Sciences, Hungarian University of Agriculture and Life Sciences, Kaposvar, Hungary; 3 Department of Soil Science, Institute of Environmental Sciences, Hungarian University of Agriculture and Life Sciences, Gödöllő, Hungary; 4 Université de Rennes, CHU de Rennes, Inserm, EHESP (école de hautes études en santé publique), Irset (Institut de recherche en santé, environnement et travail), Centre d'investigation clinique de Rennes (CIC1414), Rennes, France; 5 School of Epidemiology and Public Health, University of Ottawa and Centre for Journalology, Ottawa Hospital Research Institute, Ottawa, Canada; 6 Institute of Health Policy, Management & Evaluation, Dalla Lana School of Public Health, University of Toronto, Canada,; 7 Institut Universitaire de France, Paris, France

## Abstract

Evidence-based solutions are needed to help improve reproducibility in research. This Consensus View presents a consensus-based list of core reproducibility items for research that has been developed by a multidisciplinary group interested in research, open science, and reproducibility. The set of minimum requirements presented here outlines core expectations regarding planning, methods, data collection, management and analysis, and dissemination. This tool aims to improve the reproducibility of methods and results throughout all phases of the research process and, more generally, to promote a broader cultural shift toward transparent, reliable research.

## Abstract 2

Evidence-based solutions are needed to help improve reproducibility in research. This Consensus View presents a consensus-based list of core reproducibility items for research that has been developed by a multidisciplinary group interested in research, open science, and reproducibility.

## Introduction

Reproducibility is crucial to the progress and impact of research and innovation, as it confirms or corrects the results of studies, resulting in more reliable outcomes and a reduction in research waste [1–3]. The reproducibility of scientific results has become a proxy term for many desirable attributes of science, including reliability, replicability, and efficiency, although there is still a certain degree of variation in its definition (Box 1). Therefore, considering reproducibility in the design and conduct of research should be a precondition to establishing the quality of research. Replication studies that aim to reproduce a study or experiment are scarce, the definition of a successfully reproduced study is ambiguous, and the empirical reproducibility rate is still overall modest [4,5]. To further complicate the scenario of defining and measuring reproducibility, a recent scoping review identified 50 different metrics used to quantify, assess, explain or predict reproducibility, reflecting considerable diversity in both their nature and the specific aspects of reproducibility they address [6].

**Box 1. Definition of reproducibility and reuse**

According to the European Commission report “Assessing the reproducibility of research results in EU Framework Programmes for Research”, reproducibility is understood as a continuum from the reproduction of results (based on the same data, code, and methodology) to replication (meaning the re-enactment/repeating of results using the same analytical method, but with different datasets). These processes rely on the availability of data and methods from the original study. Thus, reproducibility relates to confirming the findings of the research (albeit with certain variations) in a similar setting.

Data reuse represents the re-analysis of a dataset or a combination of different datasets for the purpose of answering the original research questions with a new method of analysis, or answering new questions based on old data that were not necessarily the focus of the original data collection [7].

Other definitions distinguish three categories: methods reproducibility (the provision of enough detail about study procedures and data so that the same procedures could, in theory or in actuality, be exactly repeated); results reproducibility, also described as replicability (obtaining the same results from the conduct of an independent study whose procedures are as closely as possible matched to the original experiment); and inferential reproducibility (drawing qualitatively similar conclusions from either an independent replication of a study or a reanalysis of the original study) [8].

There are several reasons for the lack of reproducibility, such as technical aspects (e.g., insufficient data collection, poorly described methods, or lack of transparent reporting) and barriers in the culture and ethics of research (e.g., the need for science to be continuously innovative, pressure to publish, and career evaluations based on quantity rather than quality, as well as a lack in funding for replication of studies) [9]. Some amount of non-replicability is normal in the scientific process, inherent in certain exploratory designs or because of expected variation in sampling leading to spurious results, even when methods are appropriate and robust. Studying complex systems with imperfect knowledge and tools and deliberate choices made by researchers may also increase the occurrence of non-replicable results [10]. More often, potential reproducibility of research and the actual replication of results are hampered by limitations in the study design, its conduct, and reporting.

Adhering to reproducibility standards and best practices enables researchers to better plan their studies and helps peer reviewers assess both proposed research projects and completed outputs. These tools range from general guidance outlining key steps to enhance good research and reproducibility before, during, and after analysis [11,12], to domain-specific checklists, such as those for molecular dynamics simulations [13], artificial intelligence studies [14], clinical metabolomics research [15], or World Bank research [16]. However, most of these initiatives remain fragmented across fields and are not developed within a unified framework that could bring together diverse disciplines sharing the need for common standards. Moreover, checklists often only address computational reproducibility [17], which implies obtaining the same computational results using the same input data, computational steps, methods, code, and analysis conditions [10].

Reproducibility checks are also part of journals’ instructions and editorial process, as transparent reporting is an essential component of reproducibility. Inevitably, the assessment of methodological rigor and reliability of results stands on complete and unbiased reporting, which can be enhanced by the adoption of reporting guidelines. For example, the ARRIVE checklist for reporting animal research aims to reduce poor reporting and limit irreproducible findings that can trigger inadequate clinical studies, with risks for participants and waste of resources [18,19]. More specific examples include the reporting standards and availability of data, materials, code, and protocols at *Nature* [20], the request to fill out a reproducibility checklist along with manuscript submission at the *Journal of Artificial Intelligence Research* [21], and the American Physiological Society Rigor And Reproducibility Checklist [22]. PLOS provides general guidance for reviewers and editors to assess whether the study methods report sufficient details to allow for their replicability or reproducibility, but without specifying which items should be checked [23]. However, although reproducibility checks introduced at the publication stage may support transparent reporting, they often come too late to resolve many reproducibility-related issues [24].

To address these challenges, the Open Science to Increase Reproducibility in Science (OSIRIS) project aims to develop and test tools that improve reproducibility at various levels [25]. Here, we present a consensus-based list of core items that outlines core expectations regarding planning, methods, data collection, management and analysis, and dissemination, with the aim of increasing research reproducibility.

## The OSIRIS-Delphi study

Among the objectives of the OSIRIS project are the development and testing of tools to check compliance with reproducibility standards and best practice. Considering the increased interest in research transparency, reproducibility, and the variety of views and perspectives on how to monitor it by different interested parties, an online Delphi study was chosen as a simple and effective way to reach consensus on the core set of items to be included in a checklist for reproducibility of research projects [26,27]. The OSIRIS-Delphi study aimed at adopting a broad and global perspective, starting from a general concept of reproducibility (Box 1), that would be applicable at all stages, from planning to dissemination of a research project, and addressing the provision of sufficient details in methods to allow independent researchers to replicate an experiment and arrive at similar results.

Box 2 reports the key elements of the study methodology. Additional methodological details, including minor deviations from the protocol, are provided in the study protocol [28] and S1 File. Briefly, the Delphi study (two online surveys and one online meeting) led to the inclusion of 31 items out of the 44 items initially considered (S1 File). One item was split into two items after Round 1 (S2 File), resulting in 32 core items for reproducibility (Table 1 and S3 File). The international, multidisciplinary panel participating in this Delphi study identified these items as important for inclusion in a reproducibility checklist that would be applicable throughout the life cycle of a research project.

**Table 1:** The OSIRIS list of core items for reproducibility.

SECTION A: Planning research	
**No.**	**Title**
1	Description of the study hypotheses
2	Description of the study rationale and prior evidence (knowledge)
3	Formulation of study question(s)
4	Description of study objective(s)
5	Data management plan
6	Statistical analysis plan
**SECTION B: Material and methods**	
**No.**	**Title**
7	Description of the study population of interest
8	Description of the study sample(s)
9	Description of the materials, equipment, and other conditions of the study
10	Description of conduct and procedures
11	Description of the study variables
12	Description of measures to mitigate bias in the selection of observed objects (e.g., cells, animals, humans, data, etc.)
13	Description of measures to mitigate bias in conducting the experiment
14	Description of measures to mitigate bias in assessing outcome(s)
15	Description of measures to mitigate bias in data collection and analysis
16	Estimation of sample size before study is conducted
**SECTION C: Data collection, management, and analysis**	
**No.**	**Title**
17	Process of data collection
18	Data management (e.g., pre-processing, filtering, cleaning)
19	Data dictionary is openly available
20	Description of statistical analysis or model development and validation
21	Description of the research software or its accurate reference
22	Description of details of the software or its accurate reference along with code sharing
23	Description of applied research code (analytical tool)
**SECTION D: Dissemination**	
**No.**	**Title**
24	Tracking and reporting deviation(s) from the planned design
25	Description of failed experiments or negative or null results (if any) and documentation
26	Reporting of results in line with the planned design (i.e., the research plan or protocol)
27	Result interpretation with respect to study objectives and/or hypotheses validation
28	Reporting of strengths and limitations
29	Dataset ready for analysis is openly available or at least accessible
30	Persistent and citable identifier is assigned to dataset(s)
31	Applied research code (analytical tool) is openly available or at least accessible
32	Persistent and citable identifier is assigned to the applied research code

**Box 2. Methodology of the OSIRIS Delphi study**

**Recruitment of participants**

Participants were identified from a variety of institutions and initiatives interested in open science and reproducibility in scientific research. Recruitment aimed at covering different scientific fields, stages of research expertise, and geographic locations, but also considered the trade-offs between diversity and potential irreconcilable perspectives leading to divergent positions.

**Preliminary list of items**

The development of the preliminary list was informed by relevant literature on the general concepts of reproducibility [8,10,29] and the OSIRIS scoping review [30,31]. The list was organized into four sections (planning; materials and methods; data collection, management, and analysis; and dissemination) and designed to be agnostic across scientific disciplines. This list was translated into the Delphi Round 1 survey, the first part of which included an online consent form and a few questions to collect demographic details (including age, gender, level, and field of expertise).

**Round 1 and 2 online surveys**

During Round 1, participants were asked to rate the importance of 44 items. All the participants who completed Round 1 were then invited to participate in Round 2 and asked to rate those items that did not reach consensus in Round 1. The two online surveys were administered using LimeSurvey and lasted approximately four months in total.

**Consensus meeting**

Given the topic complexity, we planned a third-round online consensus meeting to address those items that did not reach consensus. A subgroup of the participants who completed Round 2 were identified by the OSIRIS-Delphi Steering Committee to balance gender, geographical location, research field, and stage of career, and were invited to the meeting.

**Data analysis**

We calculated the distribution of scores obtained for each item and compared them to the predefined consensus definition at each stage.

## Consensus core items for reproducibility

The OSIRIS list of checks was developed as a consensus-based tool addressing different types of reproducibility and focusing on core items that are relevant to multiple disciplines. Since reproducibility can be influenced at various stages of the scientific process, the OSIRIS list of checks was designed in a structured way, capturing distinct—though interrelated—considerations that reflect the natural progression of a research project. It is a comprehensive tool designed to highlight practices in study planning, design, analysis, and dissemination that potentially affect the reproducibility of research projects. It includes field-independent elements that are broadly, though not entirely, discipline-agnostic and applicable across research areas, distinguishes it from other available checklists. The list is intended to define the minimum requirements needed to ensure reproducibility throughout all stages of a research project’s lifecycle. To enhance usability, the checklist is divided into four sections, with related practices grouped to help researchers and other stakeholders systematically identify and address reproducibility-related issues across the different stages of a project.

### Planning research

During the planning phase, researchers need to establish a clear study design and standardized procedures through detailed and well-documented preparation. Defining protocols, methods, and analysis plans in advance can contribute to preventing bias, reduce variability, and enable researchers to repeat the study. Accordingly, this section of the list (Table 1) includes accurate descriptions of study hypotheses, rationale and prior evidence, and question(s) and objective(s), as well as the preparation of plans for data management and statistical analysis. Whenever applicable, these elements are needed for ensuring conceptual clarity and transparency, as they make explicit what the study aims to test and why. They can be achieved through early documentation of hypotheses, objectives, and analytical plans—ideally prior to data collection—while also addressing key considerations such as data management and data sharing.

Despite the agreement on these items, the group did not reach consensus on pre-registration, mainly because of different views on its applicability across types of research, e.g., hypothesis-generating or hypothesis-testing, and exploratory versus confirmatory research. The process of pre-registration (or registration, as used in clinical research) requires the definition of the research questions and analysis plan before data collection begins. Registration is considered to be a key aspect for reproducibility by many, and is already implemented as a normative practice in some fields, such as clinical research. It helps increase clarity between post-diction (the use of data to generate hypotheses) and prediction (the acquisition of data to test hypotheses), and reduces overconfidence in post-hoc explanations and selective reporting of results based on the attractiveness of findings [32]. Pre-registration is seldom adopted in disciplines outside of biomedical sciences, especially those that tend to be more exploratory and favor post-diction. Moreover, some researchers may be concerned by the alleged loss of freedom in the scientific process and the additional workload associated with the process [33,34].

### Materials and methods

The conduct phase of a project poses critically important challenges in terms of reproducibility. During this phase, planned methods and procedures are put into practice and translated into research actions, which can be influenced by a variety of factors, both internal to the research environment and external. A consistent execution requires careful description of the study materials and methods, as well as efforts to minimize bias and protocol deviations [35]. Although research is often a non-linear process, adherence to previously defined plans can ensure that the study can be repeated under the same conditions. This stage is critical for ensuring internal validity, representativeness, and minimizing analytical flexibility and bias. It can be achieved by systematically documenting sampling procedures, experimental conditions, and strategies to mitigate bias throughout the study implementation.

Accordingly, this section of the list (Table 1) includes: accurate description of the population of interest and how the study sample is built to ensure representativeness; materials, equipment, experimental conditions, steps, and variables; and measures to mitigate biases at any stage and random error.

### Data collection, management, and analysis

Proper collection, organization, and management, as well as correct interpretation of data that helps to confirm (or refute) initial study hypotheses is important especially for computational reproducibility. Rigorous data collection, management, and analytical processes are necessary to ensure the robustness and validity of study findings. This can be achieved through structured data management workflows, transparent reporting of analytical methods and software, and the provision of data dictionaries and reusable code. Therefore, this section of the list (Table 1) contains items about accurate description of data collection and management processing, statistical analysis or model development and validation, tools and software, and the availability of a data dictionary and analytical tools, research code, and software.

### Dissemination

Complete and transparent reporting of methods, data, and the results of research projects goes beyond reproducibility. It is essential for ensuring that other researchers understand what exactly was undertaken, enables error detection and self-correction, allows independent verification, and ultimately enhances credibility and trust. This section of the list (Table 1) includes items related to the alignment between plans and reporting (e.g., tracking and reporting deviation(s) from the planned design, reporting of results, and interpretation in line with study objectives or hypotheses validation) and the findability and accessibility of datasets and codes. All these aspects add to the consistency between planned and reported research verification, reuse, and cumulative knowledge building. They can be achieved by explicitly reporting deviations from initial plans and by making datasets, code, and analytical materials findable and accessible through appropriate repositories.

Concerns about reidentification and privacy in cases with sensitive data were the reasons for the lack of consensus on the sharing of raw data. Moreover, the group clearly indicated that publishing the results in open access venues or in journals with an open peer review is not a priority for reproducibility, nor were the description of authorship and contributorship.

## Challenges and future directions for the OSIRIS list of core items

Reaching consensus on a list of core items for reproducibility is an important step for future efforts in the field. However, it is important to recognize how our study characteristics may have affected the results and to what extent this tool can be adopted broadly by different users.

Although we adopted a broad and global perspective, both the development of the list of items and the process of identification and invitation of the members of the panel were inevitably influenced by the OSIRIS-Delphi Steering Committee perspective. S1 File reports the characteristics of the participants at each stage. At Round 1, 82 participants from 21 countries responded. About two-third were researchers, with a good case mix in terms of career stages. Half of the participants defined themselves as working in life sciences, but social sciences and humanities, physical sciences, and engineering were also represented. Researchers from European countries were overrepresented. These factors could have influenced the decisions to include some of the items, and we cannot exclude that different scientific communities would have selected different core items for reproducibility. However, the use of the Delphi approach assumes that consensus is a reasonable expectation, therefore some level of common interpretation of the issues under consideration was required.

**Fig 1:**
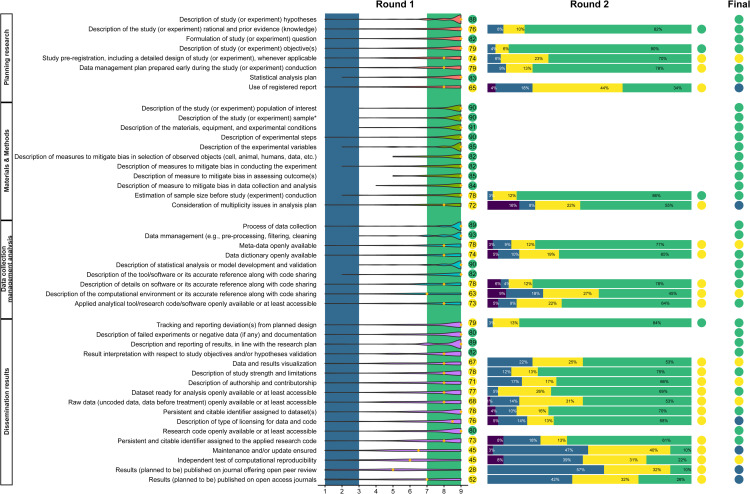
Summary of the level of consensus for each item at Rounds 1, 2, and final. **Round 1**: blue and green vertical bands correspond to the pre-defined level of agreement for exclusion (1–3) and inclusion (7–9); the violin plot (horizontal feature) corresponds to the density of answers around median values. Circles report the level of consensus, i.e., 80% of responses being in the upper third (7–9), with green = inclusion; yellow = move to Round 2. **Round 2**: green = inclusion; yellow = move to the consensus meeting; purple = no skills to answer. **Final** (after the consensus meeting): green = inclusion; yellow = no consensus; blue = exclude. The full survey at Round 1 and its revisions, a short report of the consensus meeting, anonymized data, metadata, and the statistical code are openly available on the Open Science Framework (https://osf.io/z7xmy/files).

The OSIRIS Delphi group was actively engaged, as denoted by the high response rate (91% at Round 1 and 93% at Round 2; Fig 1) and comments received (67% provided at least one comment in Round 1, 22% in Round 2). Comments received after Round 1 were not distributed before Round 2, but only before the consensus meeting; this may have limited the feedback during consensus process and was considered a weakness by some of the participants.

Sixteen panel members attended the online consensus meeting aimed at discussing the items that did not reach consensus and general themes that emerged from Round 1 and 2. Most of the comments received during the Delphi study focused on the item applicability and other general aspects about implementation. The debate on the applicability of some items to different stages of the research process, scientific field, and type (explorative, descriptive, explanatory, etc.) reflected how variable the interpretation of reproducibility can be and the set of accepted norms in each field. We recognized a degree of epistemic diversity (the systematic differences in fundamental concepts, problem formulation, empirical objects, methodological practices, and modes of judgments) across the different discipline represented [36]. From the Delphi study, the need for an implementation strategy to maximize uptake and use of these core items for reproducibility and make them an actionable and effective tool for checking reproducibility clearly emerged.

Implementation requires user testing across different research communities, as the concrete uptake of core reproducibility items depends on their interpretation and relevance for a specific stakeholder, research stage, or discipline. A collaborative pilot study that is currently underway aims to explore how researchers from diverse fields use and apply the core items recommended by the Delphi panel [37]. The first step of the pilot was a project conducted at the Responsible Research in Action Unconference (September 2025, Berlin). Researchers from various backgrounds were invited to apply the list of items to published papers in their disciplines and assess both feasibility and potential areas for adaptation. Participants appreciated the availability of a comprehensive checklist for reproducibility but reported challenges in interpreting some items—particularly those related to the planning phase, where possible overlap was noted among concepts such as study hypotheses, rationale and prior evidence, research questions, and objectives. They also observed that certain items might not be equally applicable across all types of research.

Participants highlighted that some refinements may be desirable when end-users wish to apply the checklist in their specific context. For instance, assessing whether authors “tracked and reported deviation(s) from the planned design” (item 24) would be more straightforward if pre-registration were in place—an item that did not reach consensus in the Delphi process. In the absence of pre-registration, this assessment necessarily relies on trust. They also provided valuable suggestions for revising the wording of some items that were heavily oriented toward experimental and quantitative science. A detailed description of the revised list of checks, incorporating this preliminary feedback, is available in S3 File.

The final output of the pilot phase will be an elaboration and explanation guidance to support the use of the list of checks, along with concrete examples of ideal practices. Recommendations for adapting the checks to specific disciplines and research types will also be provided, recognizing that the Delphi-derived core items were intentionally designed to address field-independent aspects of reproducibility, and that any attempt to define disciplinary agnosticism inevitably faces constraints given the diversity of scientific approaches.

Additional implementation efforts could involve editors and peer reviewers implementing the checks included in the dissemination section to assess reproducible research practices in manuscripts and provide constructive feedback to authors. Funders may find items in the planning section the most applicable to their reproducibility assessment, although other sections may be helpful in monitoring project progress. For instance, for projects funded by the European Commission, open access practices (i.e., open access to research documents and data) are required and monitored according to recommended standards [38]. Some of these reproducibility items can be embedded in this set of standards.

## Conclusions

Alongside political and economic factors, a lack of reproducibility and openness in science can undermine the trustworthiness of scientific findings and, more broadly, public confidence in the integrity of science. Tools that enable researchers and other stakeholders to enhance rigor, self-criticism, and self-correction should not be viewed as admissions of failure, but rather as mechanisms for continuous innovation [39,40].

In this Delphi study, open science and transparency across most stages of a project emerged as key drivers of reproducibility. Every research project involves a series of choices and trade-offs between rigor and feasibility. Systematically documenting decisions, assumptions, processes, data and tools—and making them visible and inspectable—can reduce undisclosed flexibility, implicit analytical steps, omissions, and dependence on tacit knowledge.

Several other checklists that have been developed in specific fields, such as molecular dynamics simulations [13], artificial intelligence [14], metabolomics [15], physiology [22], and cognitive and neuropsychological studies [41], include items concerning open science, mainly relating to data and code availability. Only the latter explicitly requires authors to state whether the study was pre-registered before data collection and analysis took place. Other tools limit this requirement to situations where they are applicable (e.g., clinical trials or systematic reviews) [12], which reflects differing views on the applicability of pre-registration across various types of research. Tools designed to promote open science and accurate and transparent reporting are also meant to increase reproducibility [5,42–45]. Generic checklists may have broad applicability but lack depth, thus potentially limiting widespread uptake. More in-depth checklists will likely only appeal to a smaller group of domain specific users. In fact, reaching consensus on what constitutes the core items for reproducibility does not in itself guarantee effectiveness, as consensus may be incomplete or overlook important aspects. Implementation studies can help determine whether these core items meaningfully identify reproducibility issues. Evidence from interventional studies, such as randomized trials currently conducted within the OSIRIS project [25], may document and quantify the impact of reproducibility checks on research practices. Meta-research studies will likely also help expand this knowledge base.

The list of items presented in this Consensus View is applicable throughout the project life cycle and can serve as a systematic framework and knowledge base for building explanation documents, guidance, and teaching materials to support researchers and other stakeholders in the proper implementation of the given items. The systematic collection of already available, and the development of new, training and education materials has a foundational role in supporting researchers and in promoting a culture of reproducibility and open science [46], but evidence is needed to assess its impact. In addition to translating the theoretical and logical construct of reproducibility into simple and feasible tools, policy, incentives, and norms to improve research credibility should be continuously aligned and updated. To promote systemic cultural change, these efforts should involve everyone involved in the research ecosystem who can support and incentivize reproducibility in a harmonized action at different organizational levels [47,48].

In this Consensus View, we aimed to identify core items for reproducibility and provide a robust starting point to support implementation efforts. We encourage the scientific community to pilot-test the checklist, refine optimal methods for its application, and—most importantly—demonstrate whether assessing these items leads to tangible improvements. Generating evidence on these practices will be essential to move beyond the status quo and reliance on wishful thinking.

## Supporting information

S1 File Details of the methodology, deviations from the protocol and characteristics of OSIRIS-Delphi Study participants. (DOCX)

S2 File Detailed results of the Delphi consensus process. (DOCX)

S3 File Detailed description of the list of checks selected by the Delphi participants. (DOCX)

## Abbreviation:

OSIRIS: Open Science to Increase Reproducibility in Science

## References

[1] Ioannidis JPA, Greenland S, Hlatky MA, Khoury MJ, Macleod MR, Moher D, et al. Increasing value and reducing waste in research design, conduct, and analysis. Lancet. 2014;383(9912):166–75. doi: 10.1016/S0140-6736(13)62227-8 24411645 PMC4697939

[2] Macleod MR, Michie S, Roberts I, Dirnagl U, Chalmers I, Ioannidis JPA, et al. Biomedical research: increasing value, reducing waste. Lancet. 2014;383(9912):101–4. doi: 10.1016/S0140-6736(13)62329-6 24411643

[3] Moher D, Glasziou P, Chalmers I, Nasser M, Bossuyt PMM, Korevaar DA, et al. Increasing value and reducing waste in biomedical research: who’s listening?. Lancet. 2016;387(10027):1573–86. doi: 10.1016/S0140-6736(15)00307-4 26423180

[4] Baker M. 1,500 scientists lift the lid on reproducibility. Nature. 2016;533(7604):452–4. doi: 10.1038/533452a 27225100

[5] Cobey KD, Fehlmann CA, Christ Franco M, Ayala AP, Sikora L, Rice DB, et al. Epidemiological characteristics and prevalence rates of research reproducibility across disciplines: a scoping review of articles published in 2018-2019. Elife. 2023;12:e78518. doi: 10.7554/eLife.78518 37341380 PMC10322148

[6] Heyard R, Pawel S, Frese J, Voelkl B, Würbel H, McCann S, et al. A scoping review on metrics to quantify reproducibility: a multitude of questions leads to a multitude of metrics. R Soc Open Sci. 2025;12(7):242076. doi: 10.1098/rsos.242076 40894102 PMC12395386

[7] European Commission. Reproducibility of scientific results in the EU – scoping report. Publications Office. 2020. Available from: https://data.europa.eu/doi/10.2777/341654

[8] Goodman SN, Fanelli D, Ioannidis JP. What does research reproducibility mean?. Sci Transl Med. 2016;8(341):341ps12. doi: 10.1126/scitranslmed.aaf5027 27252173

[9] Cobey KD, Ebrahimzadeh S, Page MJ, Thibault RT, Nguyen P-Y, Abu-Dalfa F, et al. Biomedical researchers’ perspectives on the reproducibility of research. PLoS Biol. 2024;22(11):e3002870. doi: 10.1371/journal.pbio.3002870 39499707 PMC11537370

[10] National Academies of Sciences, Engineering, Medicine. Reproducibility and replicability in science. Washington, DC: The National Academies Press. 2019. doi: 10.17226/25303 31596559

[11] Alston JM, Rick JA. A beginner’s guide to conducting reproducible research. Bull Ecol Soc Am. 2020;102(2):e01801. doi: 10.1002/bes2.1801

[12] UK Research Integrity Office. Recommended checklist for researchers 2025. Available from: https://ukrio.org/wp-content/uploads/UKRIO-Recommended-Checklist-for-Researchers.pdf

[13] Reliability and reproducibility checklist for molecular dynamics simulations. Commun Biol. 2023;6(1):268. doi: 10.1038/s42003-023-04653-0 36918708 PMC10014944

[14] Association for the Advancement of Artificial Intelligence. Reproducibility checklist. Available from: https://aaai.org/aaai-conference/reproducibility-checklist/

[15] Du X, Aristizabal-Henao JJ, Garrett TJ, Brochhausen M, Hogan WR, Lemas DJ. A checklist for reproducible computational analysis in clinical metabolomics research. Metabolites. 2022;12(1):87. doi: 10.3390/metabo12010087 35050209 PMC8779534

[16] World Bank. Checklist for World Bank Reproducibility Packages. [cited 2 Nov 2025]. Available from: https://worldbank.github.io/wb-reproducible-research-repository/reproducibility_package_checklist.html

[17] Momeni F, Khan MT, Kiesel J, Ross-Hellauer T. Checklists for computational reproducibility in social sciences: insights from literature and survey evaluation. In: Proceedings of the 3rd ACM conference on reproducibility and replicability, 2025. p. 179–91. doi: 10.1145/3736731.3746161

[18] Percie du Sert N, Ahluwalia A, Alam S, Avey MT, Baker M, Browne WJ, et al. Reporting animal research: explanation and elaboration for the ARRIVE guidelines 2.0. PLoS Biol. 2020;18(7):e3000411. doi: 10.1371/journal.pbio.3000411 32663221 PMC7360025

[19] Percie du Sert N, Hurst V, Ahluwalia A, Alam S, Avey MT, Baker M, et al. The ARRIVE guidelines 2.0: updated guidelines for reporting animal research. PLoS Biol. 2020;18(7):e3000410. doi: 10.1371/journal.pbio.3000410 32663219 PMC7360023

[20] Reporting standards and availability of data, materials, code and protocols. Nature. [cited 2 Nov 2025]. Available from: https://www.nature.com/nature/editorial-policies/reporting-standards

[21] Journal of Artificial Intelligence Research. Improving reproducibility in AI research: four mechanisms adopted by JAIR. [cited 2 Nov 2025]. Available from https://jair.org/index.php/jair/article/view/16905

[22] American Physiological Society. Rigor and reproducibility checklist. [cited 2 Nov 2025]. Available from: https://journals.physiology.org/pb-assets/PDFs/APS_Rigor-Reproducibility-Guidelines-1620307615793.pdf

[23] PLoS. Peer review checklist. [cited 2 Nov 2025]. Available from: https://plos.org/resource/peer-review-checklist/

[24] Munafò MR, Nosek BA, Bishop DVM, Button KS, Chambers CD, du Sert NP, et al. A manifesto for reproducible science. Nat Hum Behav. 2017;1(1):0021. doi: 10.1038/s41562-016-0021 33954258 PMC7610724

[25] OSIRIS Project. Open science to increase reproducibility in science. [cited 2 Nov 2025]. Available from: https://osiris4r.eu/

[26] Diamond IR, Grant RC, Feldman BM, Pencharz PB, Ling SC, Moore AM, et al. Defining consensus: a systematic review recommends methodologic criteria for reporting of Delphi studies. J Clin Epidemiol. 2014;67(4):401–9. doi: 10.1016/j.jclinepi.2013.12.002 24581294

[27] Jünger S, Payne SA, Brine J, Radbruch L, Brearley SG. Guidance on Conducting and REporting DElphi Studies (CREDES) in palliative care: recommendations based on a methodological systematic review. Palliat Med. 2017;31(8):684–706. doi: 10.1177/0269216317690685 28190381

[28] Banzi R, Naudet F, Stegeman I, Leeflang M, De Vito N, Van den Eynden V, et al. Consensus on core reproducibility checks in research: Protocol of the OSIRIS Delphi study. Open Science Foundation. 2024. [cited 2 Nov 2025]. Available from: doi: 10.17605/OSF.IO/2VGKW

[29] Munafò MR, Chambers C, Collins A, Fortunato L, Macleod M. The reproducibility debate is an opportunity, not a crisis. BMC Res Notes. 2022;15(1):43. doi: 10.1186/s13104-022-05942-3 35144667 PMC8832688

[30] Dudda L, Kormann E, Kozula M, DeVito NJ, Klebel T, Dewi APM, et al. Open science interventions to improve reproducibility and replicability of research: a scoping review. R Soc Open Sci. 2025;12(4):242057. doi: 10.1098/rsos.242057 40206851 PMC11979971

[31] Dudda LA, Kozula M, Ross-Hellauer T, Kormann E, Spijker R, DeVito N, et al. Scoping review and evidence mapping of interventions aimed at improving reproducible and replicable science: protocol. Open Res Eur. 2024;3:179. doi: 10.12688/openreseurope.16567.2 39036539 PMC11258544

[32] Nosek BA, Ebersole CR, DeHaven AC, Mellor DT. The preregistration revolution. Proc Natl Acad Sci U S A. 2018;115(11):2600–6. doi: 10.1073/pnas.1708274114 29531091 PMC5856500

[33] Evans TR, Branney P, Clements A, Hatton E. Improving evidence-based practice through preregistration of applied research: barriers and recommendations. Account Res. 2023;30(2):88–108. doi: 10.1080/08989621.2021.1969233 34396837

[34] Sarafoglou A, Kovacs M, Bakos B, Wagenmakers E-J, Aczel B. A survey on how preregistration affects the research workflow: better science but more work. R Soc Open Sci. 2022;9(7):211997. doi: 10.1098/rsos.211997 35814910 PMC9257590

[35] Isere EE, Omorogbe NE. Quality management in clinical and public health research: a panacea for minimising and eliminating protocol deviations in research operations. Niger Med J. 2024;65(3):367–75. doi: 10.60787/nmj-v65i3-421 39022564 PMC11249487

[36] Ross-Hellauer T. Strategic priorities for reproducibility reform. PLoS Biol. 2023;21(1):e3001943. doi: 10.1371/journal.pbio.3001943 36634034 PMC9836294

[37] Banzi R, Naudet F, Varga M, Vinatier C, Gelsleichter YA. Collaborative pilot of core reproducibility checks in research: applicability and implementation across scientific fields. Open Science Foundation. 2025. [cited 2 Nov 2025]. Available from: doi: 10.17605/OSF.IO/35Z2H

[38] European Research Executive Agency. Open science in Horizon Europe. [cited 2 Nov 2025]. Available from: https://rea.ec.europa.eu/open-science_en#open-science-and-project-implementation

[39] Cologna V, Mede NG, Berger S, Besley J, Brick C, Joubert M, et al. Trust in scientists and their role in society across 68 countries. Nat Hum Behav. 2025;9(4):713–30. doi: 10.1038/s41562-024-02090-5 39833424 PMC7617525

[40] Nosek BA. Science becomes trustworthy by constantly questioning itself. PLoS Biol. 2025;23(8):e3003334. doi: 10.1371/journal.pbio.3003334 40749076 PMC12327609

[41] PECANS – Preferred Evaluation of Cognitive And Neuropsychological Studies. Preferred Evaluation of Cognitive And Neuropsychological Studies - the PECANS statement for human studies. 2024. [cited 2 Nov 2025]. Available from: https://osf.io/jvze5/10.3758/s13428-025-02705-3 PMC12125112 40447861

[42] Checklists work to improve science. Nature. 2018;556(7701):273–4. doi: 10.1038/d41586-018-04590-7 30967653

[43] Aczel B, Szaszi B, Sarafoglou A, Kekecs Z, Kucharský Š, Benjamin D, et al. A consensus-based transparency checklist. Nat Hum Behav. 2020;4(1):4–6. doi: 10.1038/s41562-019-0772-6 31792401 PMC8324470

[44] Grant S, Corker KS, Mellor DT, Stewart SLK, Cashin AG, Lagisz M. TOP 2025: an update to the transparency and openness promotion guidelines. 2025. [cited 2 Nov 2025]. Available from: https://osf.io/preprints/metaarxiv/nmfs6_v2 10.1186/s41073-026-00223-0 PMC13439764 42557573

[45] Macleod M, Collings AM, Graf C, Kiermer V, Mellor D, Swaminathan S, et al. The MDAR (Materials Design Analysis Reporting) Framework for transparent reporting in the life sciences. Proc Natl Acad Sci U S A. 2021;118(17):e2103238118. doi: 10.1073/pnas.2103238118 33893240 PMC8092464

[46] Auer S, Haeltermann NA, Weissgerber TL, Erlich JC, Susilaradeya D, Julkowska M. Science Forum: a community-led initiative for training in reproducible research. eLife. 2021;10:e64719. doi: 10.7554/eLife.64719 34151774 PMC8282331

[47] Nosek BA. Strategy for culture change. 2019. [cited 2 Nov 2025]. Available from: https://www.cos.io/blog/strategy-for-culture-change

[48] Barker M, Chue Hong N. Approaches to scaling up reproducibility in research organisations. 2024. [cited 2 Nov 2025]. Available from Zenodo 10.5281/zenodo.10663903

