# Correction: An international consensus on core reproducibility items in research

**DOI:** 10.1371/journal.pbio.3003792

**Published:** 2026-05-07

**Authors:** The PLOS Biology Staff

## Notice of Republication

This article was republished on April 24, 2026, to correct errors in the acknowledgement section introduced during the proofing process. The publisher apologizes for the errors.

The acknowledgement section should read as follows:

Acknowledgments

The OSIRIS-Delphi study group are Maria Josefina Ruiz Alvarez, Ilaria Cianchetta, Nicholas J DeVito, Joanna Diong, Bjoern Gerlach, Monica Gonzalez-Marquez, Kian Hématy, Amanda Kvarven, Jan Mertens, Fakhri Momeni, Gustav Nilsonne, Ulf Toelch, Jack Wilkinson, Xu Zhou, and Mircea Zloteanu. The authors would like to thank Andreas Scherer, Enrico Glerean, Vootele Võikar, Ákos Lencsés, Sara Garofalo, Valentina Parma, Isabelle Boutron, Catherine Coirault, Eva Furrer, Daniel Stekhoven, Bernhard Voelkl, Olivia Miske, Hans Van Eyghen, Frank Ostermann, Nathalie Theret, David Colquhoun, Julien Mayor, Agata Bochynska, Tamara Kalandadze, Lewend Mayiwar, David Allison, Lars Vilhuber, Julian Lange, Josefina Weinerova, Helen Macdonald, Frits Rosendaal, Maria Panagiotopoulou, Maria Rujano, Serge Horbach, Sven Arend Ulpts, Maha Said, Tony Ross-Hellauer, Elli Papadopoulou, Arianna Caporali, Astrid Chevance, Zoè Ancion, Maria Zalm, Raphael Levy, Aki MacFarlane, Sophie Ginolhac, Alex Adams, Marco Annoni, Robert Thibault, Michael Mende, Ling Li, Anna Ambrosini, Graciana Diez-Roux, Andrew Porter, Annemieke Aartsma-Rus, Esi Aduku, Xuan Zhang, Ioana Christea, Shai Silberberg, Anna Catharina Armond, Wainer Lusoli, Randy Ellis, Ted Rohr, Vinodh Ilangovan, Peter Clayson, Alison Abritis, Kelly Cobey, Lorraine Harbison, Guillaume Sescousse, Sarah Cohen Boulakia, Esther Plomp, Adam Pollack, Jennifer Heimberg, and Jan Mertens for participating in the Delphi study. They would also like to thank Eleonora Allocati, Cinzia Colombo, Ayu Dewi, Clément Palpacuer, Priya Sharma, and Maximilian Siebert for their help in the pilot of the online survey; Maddalena Fratelli, Hynek Roubik, István Kertesz, László Baranyai, Mariska M G Leeflang, Leonie A Dudda, and Inge Stegeman for comments on the draft protocol; and Omeralfaroug Ali, Leonhard Held, István Kertész, Marianna Laviola, Jessica Rohmann, Clara Sanchez-Izquierdo Lozano, Alfredo Sanchez-Tojar, and Susanne Schorr for participating in the Responsible Research in Action Unconference.

Please download this article again to view the correct version. The originally published, uncorrected article and the republished, corrected articles are provided here for reference.

## Supporting information

S1 File Originally published, uncorrected article. (PDF)

S2 File Republished, corrected article. (PDF)
