# Supplementary material for: An international consensus on core reproducibility items in research
Source: PLoS Biol. 2026 Apr 16;24(4):e3003726. doi: 10.1371/journal.pbio.3003726 (PMC13086321; doi:10.1371/journal.pbio.3003726)
Supplement: S1 File — (DOCX) [file pbio.3003726.s001.docx]

**S1 File: details on methodology, deviations from the protocol and characteristics of OSIRIS-Delphi Study participants**

[*Study design, setting, and ethics* 1](#_Toc216112996)

[*Recruitment of participants* 1](#_Toc216112997)

[*Development of preliminary list of items* 2](#_Toc216112998)

[*Round 1 online survey* 3](#_Toc216112999)

[*Round 2 online survey* 4](#_Toc216113000)

[*Consensus meeting* 4](#_Toc216113001)

[*Data collection and analysis* 4](#_Toc216113002)

[*Deviations from the study protocol* 5](#_Toc216113003)

[*Characteristics of OSIRIS-Delphi Study participants* 6](#_Toc216113004)

[*References* 7](#_Toc216113005)

## *Study design, setting, and ethics*

The OSIRIS-Delphi Study protocol was registered on the Open Science Framework prior to data collection and approved by Rennes University Hospital ethical committee (Avis n° 24.22, Rennes, March 3rd, 2024) [1]. The study is reported following the ACCORD (Accurate Consensus Reporting Document) recommendations [2, 3].

## *Recruitment of participants*

Participants were identified from a variety of institutions and initiatives interested in open science and reproducibility in scientific research. Although we did not explicitly plan sampling criteria in terms of equity, diversity and inclusion, we aimed at including a diverse panel, individuals with a wide range of backgrounds and perspectives. Sampling aimed at covering different scientific fields, stage of research expertise, and geographic location. We primarily considered diversity in research roles and fields because we assumed these factors relate more with the question of reproducibility rather than other aspects, such as ethnicity, age, and other personal characteristics.

As described in the protocol [1], we calculated that with 80 participants the precision - defined by the boundaries of the 95% confidence interval – of the 80% agreement threshold would be ±9 percentage points.

The sampling started by contacting 46 groups including research performing organizations, scientific journal editors, research funders, and members of the public [1]. Additional potential participants were identified through snowball sampling where participants suggested other groups or people to be contacted and among the authors of papers and reports included in the OSIRIS scoping review [4, 5].

We did not collect structured information on who we contacted but did not agree to participate. Therefore, we did not include considerations on the sample representativeness compared to the target population.

The principal investigator (RB) managed the invitation by email, reminders, and finalisation of the list of participants with the support of the OSIRIS-Delphi Steering Committee (**S1 Table**).

**S1 Table: members and expertise of the OSIRIS-Delphi Study Steering Committee**

| **Member** | **Expertise** |
| --- | --- |
| Rita Banzi | Clinical pharmacology, clinical research methodology, open science |
| Florian Naudet | Therapeutics, clinical research methodology, open science |
| Monika Varga | Process modelling, model-based development of agro-food and agro-environmental processes, open science |
| Yuri Andrei Gelsleichter | Geomatics, Digital Soil Mapping, Proximal Soil Sensing, and Data Engineering |
| David Moher | Clinical epidemiology; consensus methods, open science, reproducibility, reporting guidelines |

## *Development of preliminary list of items*

The OSIRIS-Delphi Steering Committee prepared a preliminary list of items, organised in four sections (a. planning; b. materials and methods; c. data collection, management, and analysis; d. dissemination) and designed to be agnostic across scientific disciplines. This list was developed through a series of online meetings, during which key elements for reproducibility were identified, discussed, and translated into actionable checks for each stage of an ideal research project—from conception to publication, dissemination, and data-sharing provisions. This collaborative work group was informed by relevant literature on the general concepts of reproducibility [6-8] and the OSIRIS scoping review [4, 5]. The members of the OSIRIS Consortium reviewed the list, which contained 44 items to support reproducibility, briefly described to ensure clarity and standard information. This list was translated in the Round 1 survey, whose first part included an online consent form and a few questions to collect demographic details (age, gender, level and field of expertise). Six researchers not involved in the study piloted the online survey, and their feedback was used to improve the clarity of the items and their elaboration, detect technical issues concerning the platform, and estimate the average time needed to complete the survey.

Figure 1 shows the flow of participants through the study, including their identification and inclusion, and the development of the checklist items during the different Delphi rounds.

**Figure 1: Flow chart of the OSIRIS-Delphi study**


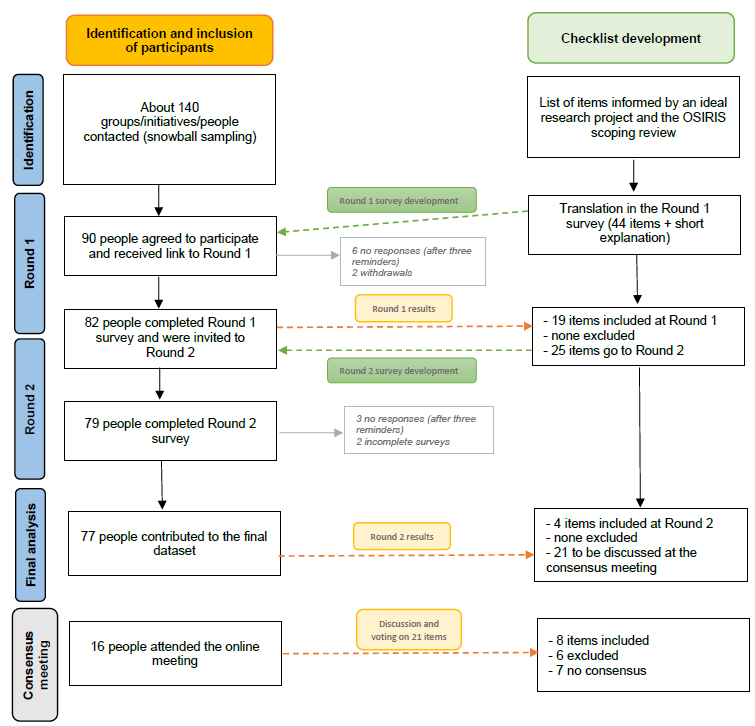


The full survey at Round 1 and its revisions are openly available on the Open Science Framework (OSIRIS Round 1 survey and items revised after Round 1 <https://osf.io/z7xmy/files>).

## *Round 1 online survey*

Participants were asked to rate the importance of each item, considering its general applicability to an ideal research project. Rating was mandatory for all the items and expressed on a 9-point Likert scale with endpoints labelled ‘Exclude this reproducibility practice from the checklist’ (1) to ‘Include this reproducibility practice from the checklist’ (9). The option “I don’t have enough content expertise to vote on this item” could be selected whenever needed. At the end of each of the four sections, participants had the chance to include comments or suggestions for additional items that they believed should be considered.

At Round 1, we defined consensus as 80% of responses being in the upper third (scores between 7-9) or bottom third (scores between 1-3) of the 9-point scale. All items in which at least 80% of the respondents indicated it should be included or excluded were removed from the voting in Round 2. We chose this threshold as it is the median value applied in 25 studies included in a systematic review in which the percentage agreement was used to determine consensus [9].

## *Round 2 online survey*

All the participants who completed Round 1 were invited to Round 2 and asked to rate those items that did not reach consensus in Round 1. Participants were first presented with the item under consideration, the aggregate group and their own scores for that item from Round 1. The addition of new items or modifications of the presented items based on qualitative feedback received after Round 1 was allowed. Participants were asked to indicate whether the item should be “included”, “excluded”, or “discussed at the consensus meeting”. The option “I don’t have expertise related to the topic’” was also available. The consensus threshold was set at 80% of the respondents who indicated the item should be included or excluded.

## *Consensus meeting*

Given the topic complexity, we planned a third-round online consensus meeting to address the items that did not reach consensus. A subgroup of all the participants who completed Round 2 was identified by the OSIRIS-Delphi Steering Committee to balance gender, geographical location, research field, and stage of career and invited to the meeting. We did not collect other information about equity, diversity, and inclusion that could have been considered for the consensus meeting invitation.

A summary of the results, including a list of pending items and key discussion topics, was distributed to attendees prior to the meeting. During this meeting, the participants were shown the result corresponding to each item, comments were invited, and then an anonymous vote was held using Mentimeter (options: include, exclude, don’t know/abstain). Consensus was defined as agreement from at least two thirds of the voters. The meeting was also aimed at discussing general themes that emerged from comments at Round 1 and 2 and gathering feedback on the strategy for implementation and testing of the checklist.

The meeting was coordinated by the principal investigator (RB), with the support of a facilitator (NJD) not directly involved in the OSIRIS-Delphi Study who moderated the discussion and the voting process.

## *Data collection and analysis*

The two online surveys were administered using LimeSurvey, a subscription-based survey tool with the necessary flexibility to adapt the checklist related questions to the need of the study. Data collection in Round 1 and 2 was estimated to last approximately four months in total.

For each step, we analysed quantitative data (i.e., number of participants and respondents, participants’ ages, and score for each item) described using the mean and standard deviation and qualitative data (gender, position, research field and aggregate themes from comments) described using frequencies and percentages. The distribution of scores obtained for each item was compared to the predefined consensus definition: consensus threshold was set at 80% of the respondents who considered the item should be included (score greater than 7) or excluded (score less than 3). All statistical analyses were done using R version 4.2.2 (RRID:SCR_001905) [10]. Rating was mandated to minimize missing responses.

Although not detailed in the study protocol, the number, complexity and heterogeneity of comments received at Round 1 required a qualitative analysis and categorization by two pairs of independent evaluators (RB, MV, CV, FN). Coding included eight categories: 1) suggestion to add new item; 2) request for clarification on specific items/concepts; 3) suggestion for reformulation/rewording of existing item; 4) comments on duplication/overlap; 5) comments on applicability; 6) support for judgment/justification of scores; 7) generic comments; 8) other. Each comment was also associated with one of the following “Action”: 1) add item; 2) reformulate item; 3) provide new explanation; 4) answer to comment; 5) interesting for the consensus meeting; 6) none. The four evaluators held virtual meetings to discuss the categorizations and reach consensus in case of disagreement.

## *Deviations from the study protocol*

Minor deviations from the protocol were deemed necessary during the study. First, the time schedule reported in the protocol was not followed because multiple waves of invitations and access to the Round 1 survey were sent out to expedite sampling. Waiting for the finalization of a complete list of participants would have led to a time gap between the agreement to participate and actual access to the survey. This also led to sending reminders to non-respondents less often than once a week.

At the end of each of the four sections, participants had the chance to include comments or suggestions for additional items that they believed should be considered. This was a deviation from the protocol, where it was planned to include only one field for comments at the end of the survey.

Although not detailed in the study protocol, the number, complexity and heterogeneity of comments received at Round 1 required a qualitative analysis.

## *Characteristics of OSIRIS-Delphi Study participants*

|  | **Round 1 (N=82)** | **Round 2 (N=77)** | **Consensus meeting (N=16)** |
| --- | --- | --- | --- |
| **Age** |  | | |
| Mean (SD) | 43.7 (11.4) | 43.6 (10.3) | 44.8 (10.1) |
| Median [Min, Max] | 42.0 [26.0, 87.0] | 42.0 [26.0, 67.0] | 42.0 [30.0, 67.0] |
| **Gender** |  |  |  |
| Female | 41 (50.0%) | 38 (49.4%) | 7 (43.8%) |
| Male | 38 (46.3%) | 36 (46.8%) | 9 (56.3%) |
| Non-binary | 1 (1.2%) | 1 (1.3%) | 0 (0%) |
| Prefer not to say | 2 (2.4%) | 2 (2.6%) | 0 (0%) |
| **Stakeholder group** |  |  |  |
| Early-career researchers (within 5 years of activities) | 16 (19.5%) | 15 (19.5%) | 1 (6.3%) |
| Mid-career researchers (5-10 years of activities) | 14 (17.1%) | 13 (16.9%) | 4 (25.0%) |
| Senior researchers (more than 10 years of activities) | 25 (30.5%) | 24 (31.2%) | 3 (18.8%) |
| Research support or administrator | 8 (9.8%) | 7 (9.1%) | 2 (12.5%) |
| Editor/publisher | 5 (6.1%) | 5 (6.5%) | 1 (6.3%) |
| Funding agency member | 7 (8.5%) | 6 (7.8%) | 2 (12.5%) |
| Policymaker | 3 (3.7%) | 3 (3.9%) | 1 (6.3%) |
| Other | 4 (4.9%)* | 4 (5.2%) | 2 (12.5%) |
| **Research Field** |  |  |  |
| Life Sciences | 48 (58.5%) | 45 (58.4%) | 9 (56.2%) |
| Social Sciences and Humanities | 18 (22.0%) | 16 (20.8%) | 2 (12.5%) |
| Physical Sciences and Engineering | 6 (7.3%) | 6 (7.8%) | 2 (12.5%) |
| Prefer not to say | 1 (1.2%) | 1 (1.3%) | 0 (0%) |
| Other | 9 (11%)** | 9 (11.7%) | 3 (18.8%) |

*One research coordinator, one employee of a non-profit science organization, two mixed.

** One journalist, one expert in metascience, one geoscience, six mixed.

## *References*

1. Banzi R, Naudet F, Stegeman I, Leeflang M, DeVito N, Van den Eynden V, et al. Consensus on core reproducibility checks in research: Protocol of the OSIRIS Delphi study, 2024. Available from Open Science Foundation, <https://doi.org/10.17605/OSF.IO/2VGKW> (access November 2, 2025).

2. Gattrell WT, Hungin AP, Price A, Winchester CC, Tovey D, Hughes EL, et al. ACCORD guideline for reporting consensus-based methods in biomedical research and clinical practice: a study protocol. Res Integr Peer Rev. 2022;7(1):3. doi: 10.1186/s41073-022-00122-0. PubMed PMID: 35672782,

3. Gattrell WT, Logullo P, van Zuuren EJ, Price A, Hughes EL, Blazey P, et al. ACCORD (ACcurate COnsensus Reporting Document): A reporting guideline for consensus methods in biomedicine developed via a modified Delphi. PLoS Med. 2024;21(1):e1004326. doi: 10.1371/journal.pmed.1004326. PubMed PMID: 38261576.

4. Dudda L, Kormann E, Kozula M, DeVito NJ, Klebel T, Dewi APM, et al. Open science interventions to improve reproducibility and replicability of research: a scoping review. R. Soc. Open Sci. 2025;12242057. doi: http://doi.org/10.1098/rsos.242057. PubMed PMID: 40206851.

5. Dudda LA, Kozula M, Ross-Hellauer T, Kormann E, Spijker R, DeVito N, et al. Scoping review and evidence mapping of interventions aimed at improving reproducible and replicable science: Protocol. Open Res Eur. 2023;3:179. doi: 10.12688/openreseurope.16567.2. PubMed PMID: 39036539.

6. Goodman SN, Fanelli D, Ioannidis JP. What does research reproducibility mean? Sci Transl Med. 2016;8(341):341ps12. doi: 10.1126/scitranslmed.aaf5027 10.1126/scitranslmed.aaf5027. PubMed PMID: 27252173.

7. Munafò MR, Chambers C, Collins A, Fortunato L, Macleod M. The reproducibility debate is an opportunity, not a crisis. BMC Res Notes. 2022;15(1):43. Epub 2022/02/12. doi: 10.1186/s13104-022-05942-3. PubMed PMID: 35144667.

8. National Academies of Sciences, Engineering, Medicine. Reproducibility and Replicability in Science. 2019. Washington, DC: The National Academies Press. https://doi.org/10.17226/25303.

9. Diamond IR, Grant RC, Feldman BM, Pencharz PB, Ling SC, Moore AM, et al. Defining consensus: a systematic review recommends methodologic criteria for reporting of Delphi studies. J Clin Epidemiol. 2014;67(4):401-9. doi: 10.1016/j.jclinepi.2013.12.002. PubMed PMID: 24581294.

10. R Core Team. A Language and Environment for Statistical Computing. Vienna, Austria: R Foundation for Statistical Computing. Available from: <https://www.R-project.org/> (access November 2, 2025).
