# Supplementary material for: An international consensus on core reproducibility items in research
Source: PLoS Biol. 2026 Apr 16;24(4):e3003726. doi: 10.1371/journal.pbio.3003726 (PMC13086321; doi:10.1371/journal.pbio.3003726)
Supplement: S2 File — (DOCX) [file pbio.3003726.s002.docx]

**S2 File: detailed results of the Delphi consensus process**

[*Round 1* 1](#_Toc211620652)

[*Round 2* 1](#_Toc211620653)

[*Consensus meeting* 2](#_Toc211620654)

[*S2 Table: List of items and level of consensus at Round 1, 2 and consensus meeting* 3](#_Toc211620655)

## *Round 1*

Participants: Eighty-two participants from 21 countries responded to Round 1 (May-July 2024, response rate 91%)

Voting: the panel reached consensus on 19 items of the 44 presented in Round 1, three in section a (planning), eight in section b (materials and methods), four in section c (data collection, management, and analysis) and four in section d (dissemination). No items were excluded, and 25 did not reach consensus and were reported in Round 2 survey. See **S2 Table**.

Qualitative analysis: Fifty-five participants (67%) provided at least one comment in Round 1. None of them led to the inclusion of new items, but several comments were used to reformulate 17 items (<https://osf.io/z7xmy/files>**)**. Most of the comments focused on the item applicability and other general aspects about implementation. For instance, *“Is this a checklist which should be employed by researchers themselves to help design their projects and make sure they are reproducible, or by others to help assess the research's reproducibility?”*; “ *I feel there are certain types of studies for which the item is not applicable or relevant (e.g. exploratory studies not based on hypotheses, conceptual studies not involving data, qualitative studies not using statistics).*”; *“Again, for many elements I would agree if the statements began "if applicable" […]. In addition, I think there are multiple elements that are definitely "nice to have" but I don’t fully see how they support reproducibility […].”*

## *Round 2*

Participants: Seventy-seven participants responded to Round 2 (September-October 2024, response rate 93%).

Voting: the panel reached consensus on four items of the 25 presented in Round 2, two in section a (planning), one in section b (materials and methods) and section c (data collection, management, and analysis) respectively. Twenty-one items did not reach consensus and were discussed in the consensus meeting. See **S2 Table.**

Qualitative analysis: Seventeen participants (22%) provided comments at Round 2. As in Round 1, comments referred to applicability and feasibility of the items across fields and type of research. Others supported the decisions on whether to include a specific item or not, for instance, “*Judging the study quality by the journal (whether it is or is not open access or has open review) is generally misleading*”. The issue of complexity and barriers to data sharing also emerged, as for instance “*The notion that data, esp. raw data, are made available is slightly complex and, whilst most of us would like this to be part of the research process negotiations are complex”.*

## *Consensus meeting*

Participants: we invited twenty-six panel members to attend the online consensus meeting (November 26 and 27, 2024) and 16 agreed to participate.

Voting: participants agreed on the inclusion of eight of the 21 items presented during the consensus meeting, one in section a (planning), three in section c (data collection, management, and analysis) and four in section d (dissemination). Six items were excluded during the consensus meeting: use of registered reports, consideration of multiplicity issues in analysis plan, description of type of licensing for data and code, maintenance and/or update of data and research software, results (planned to be) published in open access journals or journal offering open peer review.

Qualitative analysis: from the analysis of the comments received at both rounds - specifically those classified as “interesting for the consensus meeting” in the Round 1 analysis - the study Steering Committee prioritized and framed general themes used to guide the discussion during the consensus meeting: “*Who should use the checklist, at which stage?*”; “*Is a single checklist applicable across disciplines? If not, how can it be adapted to different study types, disciplines, etc.?*”; “*Can we identify core (mandatory) and additional (desirable) items?*”; “*Do we need a checklist for re-analysis of data and one for repeating experiments?*”. A report a summary of the discussion and feedback received by the participants is available on Open Science Foundation (OSIRIS-Delphi study consensus meeting short report, <https://osf.io/z7xmy/files>)

## *S2 Table: List of items and level of consensus at Round 1, 2 and consensus meeting*

|  | **Round1**  **N=82** | | **Round2**  **N=77** | | **Consensus meeting**  **N=16*** | |
| --- | --- | --- | --- | --- | --- | --- |
| **Item** | **Response** | **Frequence (%)** | **Response** | **Frequence (%)** | **Response** | **Frequence (%)** |
| Description of study (or experiment) hypotheses | ≤3 | 3 (3.7) |  |  |  |  |
|  | 4-6 | 14 (17.1) |  |  |  |  |
|  | ≥7 | 63 (76.8) |  |  |  |  |
|  | skip | 2 (2.4) |  |  |  |  |
| Description of the study (or experiment) rational and prior evidence (knowledge) | ≤3 | 6 (7.3) | Include in the checklist | 63 (81.8) |  |  |
|  | 4-6 | 25 (30.5) | Exclude from the checklist | 6 (7.8) |  |  |
|  | ≥7 | 50 (61) | To be discussed at the consensus meeting | 8 (10.4) |  |  |
|  | skip | 1 (1.2) | I don’t have expertise related to the topic | 0 (0) |  |  |
| Formulation of study (or experiment) question | ≤3 | 3 (3.7) |  |  |  |  |
|  | 4-6 | 20 (24.4) |  |  |  |  |
|  | ≥7 | 57 (69.5) |  |  |  |  |
|  | skip | 2 (2.4) |  |  |  |  |
| Description of study (or experiment) objective(s) | ≤3 | 3 (3.7) | Include in the checklist | 69 (89.6) |  |  |
|  | 4-6 | 20 (24.4) | Exclude from the checklist | 3 (3.9) |  |  |
|  | ≥7 | 56 (68.3) | To be discussed at the consensus meeting | 5 (6.5) |  |  |
|  | skip | 3 (3.7) | I don’t have expertise related to the topic | 0 (0) |  |  |
| Study pre-registration, including a detailed design of study (or experiment), whenever applicable | ≤3 | 3 (3.7) | Include in the checklist | 54 (70.1) | Include | 8 (61.5) |
|  | 4-6 | 31 (37.8) | Exclude from the checklist | 5 (6.5) | Exclude | 5 (38.5) |
|  | ≥7 | 45 (54.9) | To be discussed at the consensus meeting | 18 (23.4) | Don't know / abstain | 0 (0) |
|  | skip | 3 (3.7) | I don’t have expertise related to the topic | 0 (0) |  |  |
| Data management plan prepared early during the study (or experiment) conduction | ≤3 | 6 (7.3) | Include in the checklist | 60 (77.9) | Include | 11 (84.6) |
|  | 4-6 | 28 (34.1) | Exclude from the checklist | 7 (9.1) | Exclude | 2 (15.4) |
|  | ≥7 | 48 (58.5) | To be discussed at the consensus meeting | 10 (13) | Don't know / abstain | 0 (0) |
|  | skip | 0 (0) | I don’t have expertise related to the topic | 0 (0) |  |  |
| Statistical analysis plan | ≤3 | 2 (2.4) |  |  |  |  |
|  | 4-6 | 19 (23.2) |  |  |  |  |
|  | ≥7 | 59 (72) |  |  |  |  |
|  | skip | 2 (2.4) |  |  |  |  |
| Use of registered report | ≤3 | 8 (9.8) | Include in the checklist | 26 (33.8) | Include | 2 (15.4) |
|  | 4-6 | 27 (32.9) | Exclude from the checklist | 14 (18.2) | Exclude | 9 (69.2) |
|  | ≥7 | 42 (51.2) | To be discussed at the consensus meeting | 34 (44.2) | Don't know / abstain | 2 (15.4) |
|  | skip | 5 (6.1) | I don’t have expertise related to the topic | 3 (3.9) |  |  |
| Description of the study (or experiment) population of interest** | ≤3 | 1 (1.2) |  |  |  |  |
|  | 4-6 | 10 (12.2) |  |  |  |  |
|  | ≥7 | 70 (85.4) |  |  |  |  |
|  | skip | 1 (1.2) |  |  |  |  |
| Description of the study (or experiment) sample** | ≤3 | 1 (1.2) |  |  |  |  |
|  | 4-6 | 10 (12.2) |  |  |  |  |
|  | ≥7 | 70 (85.4) |  |  |  |  |
|  | skip | 1 (1.2) |  |  |  |  |
| Description of the materials, equipment, and experimental conditions | ≤3 | 1 (1.2) |  |  |  |  |
|  | 4-6 | 11 (13.4) |  |  |  |  |
|  | ≥7 | 69 (84.1) |  |  |  |  |
|  | skip | 1 (1.2) |  |  |  |  |
| Description of experimental steps | ≤3 | 2 (2.4) |  |  |  |  |
|  | 4-6 | 9 (11) |  |  |  |  |
|  | ≥7 | 70 (85.4) |  |  |  |  |
|  | skip | 1 (1.2) |  |  |  |  |
| Description of the experimental variables | ≤3 | 2 (2.4) |  |  |  |  |
|  | 4-6 | 12 (14.6) |  |  |  |  |
|  | ≥7 | 67 (81.7) |  |  |  |  |
|  | skip | 1 (1.2) |  |  |  |  |
| Description of measures to mitigate bias in selection of observed objects (cell, animal, humans, data, etc.) | ≤3 | 0 (0) |  |  |  |  |
|  | 4-6 | 25 (30.5) |  |  |  |  |
|  | ≥7 | 55 (67.1) |  |  |  |  |
|  | skip | 2 (2.4) |  |  |  |  |
| Description of measures to mitigate bias in conducting the experiment | ≤3 | 1 (1.2) |  |  |  |  |
|  | 4-6 | 23 (28) |  |  |  |  |
|  | ≥7 | 54 (65.9) |  |  |  |  |
|  | skip | 4 (4.9) |  |  |  |  |
| Description of measure to mitigate bias in assessing outcome(s) | ≤3 | 0 (0) |  |  |  |  |
|  | 4-6 | 22 (26.8) |  |  |  |  |
|  | ≥7 | 57 (69.5) |  |  |  |  |
|  | skip | 3 (3.7) |  |  |  |  |
| Description of measure to mitigate bias in data collection and analysis | ≤3 | 0 (0) |  |  |  |  |
|  | 4-6 | 21 (25.6) |  |  |  |  |
|  | ≥7 | 60 (73.2) |  |  |  |  |
|  | skip | 1 (1.2) |  |  |  |  |
| Estimation of sample size before study (experiment) conduction | ≤3 | 4 (4.9) | Include in the checklist | 66 (85.7) |  |  |
|  | 4-6 | 23 (28) | Exclude from the checklist | 2 (2.6) |  |  |
|  | ≥7 | 53 (64.6) | To be discussed at the consensus meeting | 9 (11.7) |  |  |
|  | skip | 2 (2.4) | I don’t have expertise related to the topic | 0 (0) |  |  |
| Consideration of multiplicity issues in analysis plan | ≤3 | 4 (4.9) | Include in the checklist | 42 (54.5) | Include | 2 (18.2) |
|  | 4-6 | 28 (34.1) | Exclude from the checklist | 6 (7.8) | Exclude | 8 (72.7) |
|  | ≥7 | 45 (54.9) | To be discussed at the consensus meeting | 17 (22.1) | Don't know / abstain | 1 (9.1) |
|  | skip | 5 (6.1) | I don’t have expertise related to the topic | 12 (15.6) |  |  |
| Process of data collection | ≤3 | 3 (3.7) |  |  |  |  |
|  | 4-6 | 8 (9.8) |  |  |  |  |
|  | ≥7 | 70 (85.4) |  |  |  |  |
|  | skip | 1 (1.2) |  |  |  |  |
| Data management (e.g., pre-processing, filtering, cleaning | ≤3 | 1 (1.2) |  |  |  |  |
|  | 4-6 | 15 (18.3) |  |  |  |  |
|  | ≥7 | 66 (80.5) |  |  |  |  |
|  | skip | 0 (0) |  |  |  |  |
| Meta-data openly available | ≤3 | 3 (3.7) | Include in the checklist | 59 (76.6) | Include | 6 (46.2) |
|  | 4-6 | 23 (28) | Exclude from the checklist | 7 (9.1) | Exclude | 4 (30.8) |
|  | ≥7 | 51 (62.2) | To be discussed at the consensus meeting | 9 (11.7) | Don't know / abstain | 3 (23.1) |
|  | skip | 5 (6.1) | I don’t have expertise related to the topic | 2 (2.6) |  |  |
| Data dictionary openly available | ≤3 | 3 (3.7) | Include in the checklist | 50 (64.9) | Include | 10 (76.9) |
|  | 4-6 | 25 (30.5) | Exclude from the checklist | 8 (10.4) | Exclude | 0 (0) |
|  | ≥7 | 51 (62.2) | To be discussed at the consensus meeting | 15 (19.5) | Don't know / abstain | 3 (23.1) |
|  | skip | 3 (3.7) | I don’t have expertise related to the topic | 4 (5.2) |  |  |
| Description of statistical analysis or model development and validation | ≤3 | 1 (1.2) |  |  |  |  |
|  | 4-6 | 12 (14.6) |  |  |  |  |
|  | ≥7 | 68 (82.9) |  |  |  |  |
|  | skip | 1 (1.2) |  |  |  |  |
| Description of the tool/software or its accurate reference along with code sharing | ≤3 | 4 (4.9) |  |  |  |  |
|  | 4-6 | 19 (23.2) |  |  |  |  |
|  | ≥7 | 57 (69.5) |  |  |  |  |
|  | skip | 2 (2.4) |  |  |  |  |
| Description of details on software or its accurate reference along with code sharing | ≤3 | 3 (3.7) | Include in the checklist | 60 (77.9) | Include | 13 (100) |
|  | 4-6 | 33 (40.2) | Exclude from the checklist | 3 (3.9) | Exclude | 0 (0) |
|  | ≥7 | 44 (53.7) | To be discussed at the consensus meeting | 9 (11.7) | Don't know / abstain | 0 (0) |
|  | skip | 2 (2.4) | I don’t have expertise related to the topic | 5 (6.5) |  |  |
| Description of the computational environment or its accurate reference along with code sharing | ≤3 | 6 (7.3) | Include in the checklist | 35 (45.5) | Include | 7 (53.8) |
|  | 4-6 | 42 (51.2) | Exclude from the checklist | 14 (18.2) | Exclude | 5 (38.5) |
|  | ≥7 | 31 (37.8) | To be discussed at the consensus meeting | 21 (27.3) | Don't know / abstain | 1 (7.7) |
|  | skip | 3 (3.7) | I don’t have expertise related to the topic | 7 (9.1) |  |  |
| Applied analytical tool/research code/software openly available or at least accessible | ≤3 | 5 (6.1) | Include in the checklist | 49 (63.6) | Include | 9 (69.2) |
|  | 4-6 | 24 (29.3) | Exclude from the checklist | 7 (9.1) | Exclude | 2 (15.4) |
|  | ≥7 | 51 (62.2) | To be discussed at the consensus meeting | 17 (22.1) | Don't know / abstain | 2 (15.4) |
|  | skip | 2 (2.4) | I don’t have expertise related to the topic | 4 (5.2) |  |  |
| Tracking and reporting deviation(s) from planned design | ≤3 | 2 (2.4) | Include in the checklist | 65 (84.4) |  |  |
|  | 4-6 | 21 (25.6) | Exclude from the checklist | 2 (2.6) |  |  |
|  | ≥7 | 57 (69.5) | To be discussed at the consensus meeting | 10 (13) |  |  |
|  | skip | 2 (2.4) | I don’t have expertise related to the topic | 0 (0) |  |  |
| Description of failed experiments or negative data (if any) and documentation | ≤3 | 3 (3.7) |  |  |  |  |
|  | 4-6 | 23 (28) |  |  |  |  |
|  | ≥7 | 56 (68.3) |  |  |  |  |
|  | skip | 0 (0) |  |  |  |  |
| Description and reporting of results, in line with the research plan | ≤3 | 3 (3.7) |  |  |  |  |
|  | 4-6 | 17 (20.7) |  |  |  |  |
|  | ≥7 | 60 (73.2) |  |  |  |  |
|  | skip | 2 (2.4) |  |  |  |  |
| Result interpretation with respect to study objectives and/or hypotheses validation | ≤3 | 3 (3.7) |  |  |  |  |
|  | 4-6 | 22 (26.8) |  |  |  |  |
|  | ≥7 | 57 (69.5) |  |  |  |  |
|  | skip | 0 (0) |  |  |  |  |
| Data and results visualization | ≤3 | 7 (8.5) | Include in the checklist | 41 (53.2) | Include | 5 (38.5) |
|  | 4-6 | 26 (31.7) | Exclude from the checklist | 17 (22.1) | Exclude | 7 (53.8) |
|  | ≥7 | 47 (57.3) | To be discussed at the consensus meeting | 19 (24.7) | Don't know / abstain | 1 (7.7) |
|  | skip | 2 (2.4) | I don’t have expertise related to the topic | 0 (0) |  |  |
| Description of study strength and limitations | ≤3 | 5 (6.1) | Include in the checklist | 58 (75.3) | Include | 10 (76.9) |
|  | 4-6 | 21 (25.6) | Exclude from the checklist | 9 (11.7) | Exclude | 3 (23.1) |
|  | ≥7 | 55 (67.1) | To be discussed at the consensus meeting | 10 (13) | Don't know / abstain | 0 (0) |
|  | skip | 1 (1.2) | I don’t have expertise related to the topic | 0 (0) |  |  |
| Description of authorship and contributorship | ≤3 | 6 (7.3) | Include in the checklist | 51 (66.2) | Include | 8 (61.5) |
|  | 4-6 | 28 (34.1) | Exclude from the checklist | 13 (16.9) | Exclude | 4 (30.8) |
|  | ≥7 | 47 (57.3) | To be discussed at the consensus meeting | 13 (16.9) | Don't know / abstain | 1 (7.7) |
|  | skip | 1 (1.2) | I don’t have expertise related to the topic | 0 (0) |  |  |
| Dataset ready for analysis openly available or at least accessible | ≤3 | 3 (3.7) | Include in the checklist | 53 (68.8) | Include | 12 (92.3) |
|  | 4-6 | 26 (31.7) | Exclude from the checklist | 4 (5.2) | Exclude | 0 (0) |
|  | ≥7 | 52 (63.4) | To be discussed at the consensus meeting | 20 (26) | Don't know / abstain | 1 (7.7) |
|  | skip | 1 (1.2) | I don’t have expertise related to the topic | 0 (0) |  |  |
| Raw data (uncoded data, data before treatment) openly available or at least accessible | ≤3 | 2 (2.4) | Include in the checklist | 41 (53.2) | Include | 6 (46.2) |
|  | 4-6 | 33 (40.2) | Exclude from the checklist | 11 (14.3) | Exclude | 5 (38.5) |
|  | ≥7 | 43 (52.4) | To be discussed at the consensus meeting | 24 (31.2) | Don't know / abstain | 2 (15.4) |
|  | skip | 4 (4.9) | I don’t have expertise related to the topic | 1 (1.3) |  |  |
| Persistent and citable identifier assigned to dataset(s) | ≤3 | 2 (2.4) | Include in the checklist | 54 (70.1) | Include | 11 (84.6) |
|  | 4-6 | 27 (32.9) | Exclude from the checklist | 8 (10.4) | Exclude | 1 (7.7) |
|  | ≥7 | 51 (62.2) | To be discussed at the consensus meeting | 12 (15.6) | Don't know / abstain | 1 (7.7) |
|  | skip | 2 (2.4) | I don’t have expertise related to the topic | 3 (3.9) |  |  |
| Description of type of licensing for data and code | ≤3 | 5 (6.1) | Include in the checklist | 52 (67.5) | Include | 3 (27.3) |
|  | 4-6 | 23 (28) | Exclude from the checklist | 11 (14.3) | Exclude | 7 (63.6) |
|  | ≥7 | 52 (63.4) | To be discussed at the consensus meeting | 10 (13) | Don't know / abstain | 1 (9.1) |
|  | skip | 2 (2.4) | I don’t have expertise related to the topic | 4 (5.2) |  |  |
| Research code openly available or at least accessible | ≤3 | 1 (1.2) |  |  |  |  |
|  | 4-6 | 21 (25.6) |  |  |  |  |
|  | ≥7 | 58 (70.7) |  |  |  |  |
|  | skip | 2 (2.4) |  |  |  |  |
| Persistent and citable identifier assigned to the applied research code | ≤3 | 3 (3.7) | Include in the checklist | 47 (61) | Include | 9 (69.2) |
|  | 4-6 | 30 (36.6) | Exclude from the checklist | 14 (18.2) | Exclude | 0 (0) |
|  | ≥7 | 45 (54.9) | To be discussed at the consensus meeting | 10 (13) | Don't know / abstain | 4 (30.8) |
|  | skip | 4 (4.9) | I don’t have expertise related to the topic | 6 (7.8) |  |  |
| Maintenance and/or update ensured | ≤3 | 9 (11) | Include in the checklist | 8 (10.4) | Include | 3 (23.1) |
|  | 4-6 | 40 (48.8) | Exclude from the checklist | 36 (46.8) | Exclude | 10 (76.9) |
|  | ≥7 | 25 (30.5) | To be discussed at the consensus meeting | 31 (40.3) | Don't know / abstain | 0 (0) |
|  | skip | 8 (9.8) | I don’t have expertise related to the topic | 2 (2.6) |  |  |
| Independent test of computational reproducibility | ≤3 | 11 (13.4) | Include in the checklist | 17 (22.1) | Include | 4 (36.4) |
|  | 4-6 | 47 (57.3) | Exclude from the checklist | 30 (39) | Exclude | 7 (63.6) |
|  | ≥7 | 20 (24.4) | To be discussed at the consensus meeting | 24 (31.2) | Don't know / abstain | 0 (0) |
|  | skip | 4 (4.9) | I don’t have expertise related to the topic | 6 (7.8) |  |  |
| Results (planned to be) published on journal offering open peer review | ≤3 | 22 (26.8) | Include in the checklist | 8 (10.4) | Include | 1 (7.7) |
|  | 4-6 | 44 (53.7) | Exclude from the checklist | 44 (57.1) | Exclude | 10 (76.9) |
|  | ≥7 | 14 (17.1) | To be discussed at the consensus meeting | 25 (32.5) | Don't know / abstain | 2 (15.4) |
|  | skip | 2 (2.4) | I don’t have expertise related to the topic | 0 (0) |  |  |
| Results (planned to be) published on open access journals | ≤3 | 19 (23.2) | Include in the checklist | 20 (26) | Include | 1 (7.7) |
|  | 4-6 | 27 (32.9) | Exclude from the checklist | 32 (41.6) | Exclude | 11 (84.6) |
|  | ≥7 | 36 (43.9) | To be discussed at the consensus meeting | 25 (32.5) | Don't know / abstain | 1 (7.7) |
|  | skip | 0 (0) | I don’t have expertise related to the topic | 0 (0) |  |  |

* Not all the attendees at the consensus meeting voted to all questions. Frequencies are calculated on the actual number of voters (11 or 13).

** Included as single item at Round 1 (Description of the study (or experiment) population) and then split in two
