# Supplementary material for: An international consensus on core reproducibility items in research
Source: PLoS Biol. 2026 Apr 16;24(4):e3003726. doi: 10.1371/journal.pbio.3003726 (PMC13086321; doi:10.1371/journal.pbio.3003726)
Supplement: S3 File — (DOCX) [file pbio.3003726.s003.docx]

**S3 File_detailed description of the list of checks selected by the Delphi participants^[[1]](#footnote-1)^**

| **SECTION A: Planning research** | | |
| --- | --- | --- |
| **No.** | **Title** | **Description and elaboration (why the item is important for reproducibility)** |
| 1 | Description of study hypotheses | The study hypothesis is a clear statement of testable expectation or prediction based on earlier observation or theory, that will be tested by the research project.  In the scientific method, an experiment is an empirical procedure that arbitrates competing models or hypotheses [1, 2]. Research projects may describe a phenomenon or test existing theories or explore new hypotheses to support or disprove them [2, 3]. Progress in science relies in part on generating hypotheses with existing observations and testing them with new observations. In the scientific method, the hypothesis is constructed before any applicable research has been done, apart from a basic background review. In the scientific workflow, it can either precede or follow the formulation of the research question(s) [4]. |
| 2 | Description of the study rationale and prior evidence (knowledge) | The rationale is the justification for taking on a given research project that should be based on the prior evidence (knowledge) as background.  The rationale explains the hypothesis and reason why the study (research) project should be conducted, and it addresses the knowledge gap, prior to the execution. Usually, the rationale of the study links the (literature) background to the specific research question and justifies the need for the latter based on the former. The rationale defines how the study (research) question and objectives are framed [5]. |
| 3 | Formulation of study question(s) | The study question is the primary question(s) that a research project sets out to answer. Most research-focused questions lead to studies that aim to generate new insights [6-8]. |
| 4 | Description of study objective(s) | Study objectives define the specific actions to achieve the aims of the study, and they are usually stated in the introduction of the research protocol or, commonly, in the last parts of the introduction in scientific articles [5]. The study objective(s) usually derive from the hypothesis. |
| 5 | Data management plan | Data management plans detail how data will be collected, processed, analysed, described, preserved, and shared during a research project.  Data management plan that is associated with a research study must include structured and comprehensive information about the data such as the types of data produced, the metadata standards used, the policies for access and sharing, and the plans for archiving and preserving data so that it is accessible over time. Data management plans ensure that data will be properly documented and include details to make data FAIR (Findable, Accessible, Interoperable, Reusable) [9]. Best practice is to develop *a priori* data management plans before data collection and analysis has started. Data management plans are not usually included in research articles but may be referenced and available for inspection. |
| 6 | Statistical analysis plan | Statistical analysis plan describes the planned analysis of study objectives, what variables and outcomes will be collected, and which statistical methods will be used to analyse them.  Statistical analysis plan supplements the protocol and provides richer detail for all planned statistical analyses. In addition, it defines the population(s) and time point(s) used for each analysis, measures for multiplicity control and for handling missing data, sensitivity and subset analyses prospectively identified [10]. Best practice is to develop an *a priori* statistical analysis plan, i.e. at the time of protocol development, and finalize it prior to database lock and statistical analysis [11]. With few exceptions, statistical analysis plans are not usually included in research articles but may be referenced and available for inspection. |
| **SECTION B: Material and Methods** | | |
| **No.** | **Title** | **Description and elaboration (why the item is important for reproducibility)** |
| 7 | Description of the study population of interest | The population of interest refers to the group a researcher wants to study and draw conclusions or inferences about [12].  The population of interest is the entire group in a research context to which the findings may be applied (i.e., generalizability or transferability) [13, 14]. It could include organisms, materials, datasets, or systems depending on the field. |
| 8 | Description of the study sample(s) | Sample(s) are subsets of the target population, obtained through the process of sampling, that is how to select the group from which data are collected [13-15]. For instance, biological samples, materials, data, articles, etc.  When applicable, the sample(s) may include positive and negative controls used to assess the test validity of the experimental protocol or equipment. |
| 9 | Description of the materials, equipment, and other conditions of the study | It broadly refers to the tools (physical or digital) used in a study (research project). For instance, reagents, videos, equipment, permissions, study areas, software, workflow, etc. In clinical research, it may include the description of the study intervention and co-interventions and settings; in preclinical research, laboratory instruments and conditions, as well as measures to address animal welfare issues; in agro-environmental research, managerial practices (to study real-world systems) and external experimental factors (temperature, radiation, etc.) [16]. |
| 10 | Description of conduct and procedures | It refers to the description of how the sequence of the research process is organized and controlled to reach the study objective(s) [17]. |
| 11 | Description of the study variables | A variable is defined as an independent or dependent variable, central to the investigation, that is manipulated by the researcher to determine its relationship to or influence upon some outcome or dependent variable [13]. |
| 12 | Description of measures to mitigate bias in selection of observed objects (cell, animal, humans, data, etc.) | It usually refers to strategies that reduce the potential for selecting biased samples across a population(s). In certain cases, systematic errors arise due to differences between objects selected for study and those not selected.  If individuals or groups in a study differ systematically from the population of interest, leading to a systematic error in an association or outcome. For instance, if a study relies on volunteers, these tend to be qualitatively different from those who do not volunteer for several reasons. It also refers to ascertainment bias, that implies systematic differences in the identification of objects included in a study or distortion in the collection of data in a study. Random sampling and randomization are mitigation strategies for selection and ascertainment [18]. |
| 13 | Description of measures to mitigate bias in conducting the study | It refers to measures employed to prevent bias during the conduction of a study (research project).  Certain issues like systematic differences can emerge during the conduction, with deviations from how it was planned that are driven by specific characteristics of the study population, as for instance group assignment. E.g. in clinical studies, performance bias leads to systematic differences in the care provided to members of different study groups other than the intervention under investigation [18]. Blinding of study personnel and study objects (when applicable) and other specific measures are suggested in several fields of life sciences [19]. |
| 14 | Description of measures to mitigate bias in assessing outcome(s) | It refers to how outcome measurements were protected from bias.  Errors in measuring the outcome variables arise when the measured values do not equal the true or underlying values and can bias estimates of the effect. These errors include measurement errors, misclassifications, and under-ascertainment/over-ascertainment. Biases are usually due to differential measurement errors, i.e. related to specific characteristics of the study population, for instance group assignment. Consideration of risk of bias in this domain depends on whether the method of measuring the outcome is appropriate; measurement or ascertainment of the outcome could differ or be influenced by who the outcome assessor is, and whether outcome assessors were blinded to experimental conditions [20]. Blinding of outcome assessors can mitigate the influence of researcher's perspective on the results of a study. |
| 15 | Description of measures to mitigate bias in data collection and analysis | Data collection and analysis can be biased because of intended distortion, i.e. related to specific characteristics of the study population, as for instance group assignment. Blinding statisticians and other personnel in charge of data analysis may limit this bias [21]. |
| 16 | Estimation of sample size before study conduction | Sample size refers to the number of objects planned to be included in the study.  Most of the research is conducted on samples because it is usually impossible to study the entire population of interest. The sample must be adequate in size, as a larger sample may be unnecessary, unfeasible, and unethical, and a very small sample size may lead to poor precision and may be difficult to interpret [22, 23]. Whenever applicable, sample size calculation or power analysis needs to state assumptions, the method used, and the source of estimates for effect size. |
| **SECTION C: Data collection, management and analysis** | | |
| **No.** | **Title** | **Description and elaboration (why the item is important for reproducibility)** |
| 17 | Process of data collection | Data collection refers to the process of gathering and measuring information on variables of interest, in an established systematic form that enables one to answer stated research questions, test hypotheses, and evaluate outcomes. The data collection component of research is common to all fields of study, including physical and social sciences, humanities, business, etc. |
| 18 | Data management (e.g., pre-processing, filtering, cleaning) | Data management is supported by a structured description of data acquisition/collection, analysis, management, storage and curation during a research project. It can also refer to technical details of processing, data ownership and sharing/preserving. It includes data pre-processing, e.g. the preparation of dataset(s) for the subsequent detailed analysis (check of missing values and how to handle them, noisy or other inconsistent data, error handling and exceptions). Data management may also include proper citation of external datasets re-used for the purposes of the study investigation [24, 25]. |
| 19 | Data dictionary openly available | A data dictionary is a document that outlines the structure, content, and meaning of a given variable. A data dictionary is used to catalogue and communicate the structure and content of data and provides meaningful descriptions for individually named data objects. This includes what type of data is being collected (e.g. free text, numerical, categorical, or group data), the full wording of a question, what values are allowable (e.g. numeric ranges, multiple choice codes), and what those values mean (e.g. 0 = no high blood pressure diagnosis, 1 = borderline high blood pressure, 2 = high blood pressure) [26]. README Files can be interpreted as a form of documentation that explains the dataset at a granular level (e.g. the content and structure of a folder and/or variables, their acceptable ranges, sample values, etc.,) so that a researcher can locate the information they need. |
| 20 | Description of statistical analysis or model development and validation | Statistical analysis means investigating trends, patterns, and relationships using (quantitative) data. Data analysis comprises data description, analytical methods and tools defined by the study objectives. Data analysis can be descriptive, exploratory, inferential, predictive, explanatory or causal, mechanistic and may include testing positive and negative controls [27]. Model development, for instance, computational models for prediction, requires an iterative process in which many models are derived, tested and built upon until a model fits the desired criteria. The procedure for developing and using a model of a system involves three steps: sketching the conceptual model, refining it, and developing an appropriately identified and validated detailed model [28]. Complex models, such as machine learning models, require validation. Validation methods include leave-one -out cross-validation, k-fold cross-validation, and external validation, among others. |
| 21 | Description of the research software or its accurate reference | Software for data analysis is an integral component of the research process; it can serve, e.g., as a component of a physical or virtual instrument to analyse research data, present research results, assemble or integrate existing components into a working whole, or be the infrastructure itself [29]. Research software is defined as a software that “solves complex modelling problems in a scientific context (physics, mathematics, biology, medicine, social science, neuroscience, engineering); supports the functioning of research instruments or the execution of research experiments; extracts knowledge from large data sets; offers a mathematical library, or similar” [30]. Research software may be used to capture data and perform complex analysis with reports, graphs, and image-generating facilities for the scientific community. |
| 22 | Description of details on software or its accurate reference along with code sharing | In context of the previous item, proper description or citation of the software used for the given research work is important to understand, reproduce or re-use studies where software is an integral part of the research process. Details include information about licensing and definition of system and user requirements (platform, version, dependencies, applied libraries). When reference is made to existing software shortcut citations without a complete description in the referenced paper should be avoided. In the case of commercial tool, the references should include licensing information, necessary credentials or instructions for use [31]. |
| 23 | Description of applied research code (analytical tool) | Research code (or analytical tool) is a computer code (script) written to generate, process, analyse and validate research data (mostly not compiled code). Research code (or analytical tool) are usually developed within a scientific software using a range of programming languages and often employ specialized development environments and tools (IDEs, notebooks, version control) to enable collaboration, reproducibility, and rigorous scientific analysis. |
| **SECTION D: Dissemination of results** | | |
| **No.** | **Title** | **Description and elaboration (why the item is important for reproducibility)** |
| 24 | Tracking and reporting deviation(s) from planned design | Adherence to the planned design, for instance as reported in the study protocol, may not always be feasible and often is not even desirable. Minor differences are usually referred to as “deviation”, while divergence that affects the quality of data and the study itself is classified as “violation” [32]. If a protocol is available, tracking and documenting deviations and violations may help to assess to what extent the study was conducted as planned [33]. |
| 25 | Description of failed experiments or negative or null results (if any) and documentation | Negative results are those findings that fail to support the study hypothesis, often by showing no significant effect or an opposite one. While sometimes seen as failures, these results are crucial for scientific progress as they can correct incorrect hypotheses, prevent other researchers from wasting time and resources and help refine existing knowledge and promote better and more accurate interpretations of scientific investigations [34]. |
| 26 | Reporting of results in line with the planned design, i.e. research plan, protocol | Research results should be published as planned – ideally in the study protocol - otherwise there is a failure to adequately inform research users, including patients and the broader public. Apart from missing data, it is widely known that non-reporting is more frequent when results are statistically negative or show no effect. This phenomenon, known as outcome reporting bias, occurs when authors fail to report unfavourable data, include only a subset of data analysed, or change or omit the outcome of interest to achieve statistical significance [18, 35-37]. Absence of a protocol can undermine the rigor of a study and affect the ability to assess reporting bias. Inconsistencies between study methods and results, discrepancies or any non-standard or unusual reporting of outcomes may signal reporting bias. |
| 27 | Result interpretation with respect to study objectives and/or hypotheses validation | Data interpretation refers to the method of using various analytical processes in which data turns into useful information. This involves asking a series of questions about data that relates to the original study questions/hypotheses. Answers to these questions are organized as findings and conclusions that give a basis for recommendation. In this regard, interpretation must distinguish between directly obtained results and speculative insights [38]. |
| 28 | Reporting strength and limitations | The description of study strength and the disclosure of research limitations can help the reader better understand the conditions surrounding the study and the challenges the researcher has encountered. Limitations commonly emerge from inappropriate design, data collection and/or analytical methods and should be reported in full. The limitations of any research study will be rooted in the validity of its results—specifically threats to internal or external validity [39]. Some limitations may have a greater impact on reproducibility. |
| 29 | Dataset ready for analysis openly available or at least accessible | After collection (and assessment, abstraction, or adjudication as appropriate), source data typically must be entered into an organized data management system (i.e., database/trusted repository) for further evaluation and processing. Data typically undergo a process of cleaning, quality assurance, and quality control to detect inconsistent, incomplete, or inaccurate entries, and to confirm that the data were collected and evaluated according to the protocol and that they match the source data. This process continues throughout the course of the investigation as data is collected. After data are entered in computerized form, new variables can be mathematically generated to serve as the basis for later analyses. These variables are sometimes called “derived” variables. For example, patient age might not be entered directly but calculated by subtracting the birthdate from the date of a given clinic visit [40]. Accessibility of datasets is encouraged to be documented with minimal or rich metadata in line with the FAIR principles and respectful of legal and ethical limitations, when applicable. |
| 30 | Persistent and citable identifier assigned to dataset(s) | Persistent identifiers (as the digital object identifier, DOI) are used to uniquely identify objects, such as text documents and research data and make them permanently findable and citable. Identifiers are a pillar of the FAIR principles (Findability, Accessibility, Interoperability, and Reuse of digital assets). Findability: The first step in (re)using data is to find them. Metadata and data should be easy to find for both humans and computers. Machine-readable metadata are essential for automatic discovery of datasets and services, so this is an essential component of the FAIRification process [41]. |
| 31 | Applied research code (analytical tool) openly available or at least accessible | Accessibility of research code (or analytical tool) and research software in line with the FAIR principles [41] assists other researchers to evaluate, reuse and replicate research studies.  Research code should be openly available or accessible with an open licence and/or third-party software cited by the researcher [42]. Ideally, research code is openly available with an open licence where possible. The applied research code (analytical tool) and software are open source when the users have the freedom to run, copy, distribute, study, change and improve the software, while it is also downloadable and accessible without any charge. |
| 32 | Persistent and citable identifier assigned to the applied research code | Persistent identifiers (as the digital object identifier, DOI) are used to uniquely identify objects, such as text documents and research data and make them permanently findable and citable. Identifiers are a pillar of the FAIR principles (Findability, Accessibility, Interoperability, and Reuse of digital assets). Findability: The first step in (re)using data is to find them. Metadata and data should be easy to find for both humans and computers. Machine-readable metadata are essential for automatic discovery of datasets and services, so this is an essential component of the FAIRification process [41]. |



**References**

1. Cooperstock FI. General Relativistic Dynamics: Extending Einstein's Legacy Throughout the Universe: World Scientific; 2009.

2. Griffith WT, Brosing JW. The Physics of Everyday Phenomena: A Conceptual Introduction to Physics: McGraw-Hill; 2021.

3. Wilczek F. Fantastic Realities: 49 Mind Journeys And A Trip To Stockholm: World Scientific Publishing Company; 2006.

4. Farrugia P, Petrisor BA, Farrokhyar F, Bhandari M. Practical tips for surgical research: Research questions, hypotheses and objectives. Can J Surg. 2010;53(4):278-81.

5. American Journal Experts. How to Write the Rationale for a Research Paper. Available from: <https://www.aje.com/arc/how-to-write-the-rationale-for-a-research-paper/> Accessed October 29, 2025.

6. Haynes B. Forming research questions. J Clin Epidemiol. 2006;59(9):881-6.

7. Hulley SB, Cummings SR, Browner WS, Grady DG, Newman TB. Designing Clinical Research: Wolters Kluwer Health; 2013.

8. Mattick K, Johnston J, de la Croix A. How to…write a good research question. Clin Teach. 2018;15(2):104-8.

9. Parsons S, Azevedo F, Elsherif MM, Guay S, Shahim ON, Govaart GH, et al. A community-sourced glossary of open scholarship terms. Nature Human Behaviour. 2022;6(3):312-8.

10. Stevens G, Dolley S, Mogg R, Connor JT. A template for the authoring of statistical analysis plans. Contemporary Clinical Trials Communications. 2023;34:101100.

11. Petersen KS, Kris-Etherton PM, McCabe GP, Raman G, Miller JW, Maki KC. Perspective: Planning and Conducting Statistical Analyses for Human Nutrition Randomized Controlled Trials: Ensuring Data Quality and Integrity. Adv Nutr. 2021;12(5):1610-24.

12. Dovedetail. What is a population of interest? Available from: <https://dovetail.com/research/population-of-interest/> Accessed October 29, 2025.

13. American Psychology Association Dictionary of Psychology. Available from: <https://dictionary.apa.org/> Accessed October 29, 2025.

14. Kukull WA, Ganguli M. Generalizability: the trees, the forest, and the low-hanging fruit. Neurology. 2012;78(23):1886-91.

15. Melo SM, Carver JC, Souza PSL, Souza SRS. Empirical research on concurrent software testing: A systematic mapping study. Information and Software Technology. 2019;105:226-51.

16. Diggle PJ, Chetwynd A. Statistics and Scientific Method: An Introduction for Students and Researchers: OUP Oxford; 2011.

17. Bell S. Experimental Design. In: Kitchin R, Thrift N, editors. International Encyclopedia of Human Geography. Oxford: Elsevier; 2009. p. 672-5.

18. Catalogue of bias: centre for evidence-based medicine, Oxford University. Available from: <https://catalogofbias.org/biases/> Accessed October 29, 2025.

19. Holman L, Head ML, Lanfear R, Jennions MD. Evidence of Experimental Bias in the Life Sciences: Why We Need Blind Data Recording. PLoS Biol. 2015;13(7):e1002190.

20. Sterne JAC, Savović J, Page MJ, Elbers RG, Blencowe NS, Boutron I, et al. RoB 2: a revised tool for assessing risk of bias in randomised trials. BMJ. 2019;366:l4898.

21. Marques J. Understanding and Mitigating Data Bias in Data Analysis and Data Science 2023 Available from: <https://medium.com/@marquesjef/understanding-and-mitigating-data-bias-in-data-analysis-and-data-science-15a5e9282356> Accessed October 29, 2025.

22. Fletcher J. Sample sizes. BMJ. 2008;337:a199.

23. Sedgwick P. Units of sampling, observation, and analysis. BMJ. 2015;351:h5396.

24. Framework for Open and Reproducible Research Training (Forrt). Data Management Plans 2024 Available from: <https://forrt.org/tag/data-management-plans/> Accessed October 29, 2025.

25. National Institute of Health. Data Management Plan. Available from: <https://www.nnlm.gov/guides/data-glossary/data-management-plan> Accessed October 29, 2025.

26. Open Science Foundation. How to make a data dictionary. Available from: <https://help.osf.io/article/217-how-to-make-a-data-dictionary> Accessed October 29, 2025.

27. Taherdoost H. Different Types of Data Analysis; Data Analysis Methods and Techniques in Research Projects Authors. 2020.

28. Parush A. Conceptual Design for Interactive Systems: Designing for Performance and User Experience: Morgan Kaufmann; 2015.

29. van Nieuwpoort R, Katz DS. Defining the roles of research software. Available from: <https://upstream.force11.org/defining-the-roles-of-research-software/> Accessed October 29, 2025.

30. Journal of Open Source Software (JOSS). Submitting a paper to JOSS. Available from: <https://joss.readthedocs.io/en/latest/submitting.html#what-we-mean-by-research-software> Accessed October 29, 2025.

31. Jackson M. How to cite and describe software. Available from: <https://www.software.ac.uk/publication/how-cite-and-describe-software> Accessed October 29, 2025.

32. Bhatt A. Protocol deviation and violation. Perspectives in Clinical Research. 2012;3(3).

33. Claesen A, Gomes S, Tuerlinckx F, Vanpaemel W. Comparing dream to reality: an assessment of adherence of the first generation of preregistered studies. R Soc Open Sci. 2021;8(10):211037.

34. Matosin N, Frank E, Engel M, Lum JS, Newell KA. Negativity towards negative results: a discussion of the disconnect between scientific worth and scientific culture. Dis Model Mech. 2014;7(2):171-3.

35. EQUATOR Network: Enhancing the QUAlity and Transparency Of health Research. Available from: <https://www.equator-network.org/> Accessed October 29, 2025.

36. Geospatial Analysis for Optimization at Environmental Sites. Available from: <https://gro-1.itrcweb.org/documenting-results/> Accessed October 29, 2025.

37. Kirkham JJ, Altman DG, Chan AW, Gamble C, Dwan KM, Williamson PR. Outcome reporting bias in trials: a methodological approach for assessment and adjustment in systematic reviews. BMJ. 2018;362:k3802.

38. Tulsai K. What is Data Interpretation and How to Interpret Data Efficiently 2022 Available from: <https://medium.com/@karan_19206/what-is-data-interpretation-and-how-to-interpret-data-efficiently-b0519a69c45a> Accessed October 29, 2025.

39. Ross PT, Bibler Zaidi NL. Limited by our limitations. Perspect Med Educ. 2019;8(4):261-4.

40. IOM (Institute of Medicine). 2014. Discussion framework for clinical trial data sharing: Guiding principles, elements, and activities. Washington, DC: The National Academies Press.

National Academies of Sciences, Engineering, and Medicine. 2014. Discussion Framework for Clinical Trial Data Sharing: Guiding Principles, Elements, and Activities. Washington, DC: The National Academies Press. Available from: <https://doi.org/10.17226/18610> Accessed October 29, 2025.

41. Fair. F1: (Meta) data are assigned globally unique and persistent identifiers. Available from: <https://www.go-fair.org/fair-principles/f1-meta-data-assigned-globally-unique-persistent-identifiers/> Accessed October 29, 2025.

42. PLOS Computational Biology: code availability. Available from: <https://journals.plos.org/ploscompbiol/s/code-availability> Accessed October 29, 2025.

1. *In this context, the term “studies” should be interpreted in a broad sense, encompassing diverse forms of research project or investigation across various scientific disciplines. It is applicable to research aimed at “gaining insights” or “extracting meaning”, sometimes referred to as descriptive and analytical for prediction and counterfactual prediction (causality), or exploratory and confirmatory research. Moreover, it should be intended as covering research on both quantitative and qualitative data generation or analysis.*

   *The item description and elaboration are reported as developed for the Delphi study and revised to account for comments and suggestions received by the RRiA Unconference participants. Further development of the item description and elaboration is ongoing.* [↑](#footnote-ref-1)
